# A Real-Time Wireless Sweat Rate Measurement System for Physical Activity Monitoring

**DOI:** 10.3390/s18020533

**Published:** 2018-02-10

**Authors:** Andrew Brueck, Tashfin Iftekhar, Alicja B. Stannard, Kumar Yelamarthi, Tolga Kaya

**Affiliations:** 1 School of Engineering and Technology, Central Michigan University, Mt Pleasant, MI 48859, USA; bruec1ap@cmich.edu (A.B.); iftek1a@cmich.edu (T.I.); yelam1k@cmich.edu (K.Y.); 2 Department of Physical Therapy and Human Movement Science, Sacred Heart University, Fairfield, CT 06825, USA; stannarda@sacredheart.edu; 3 School of Computing, Sacred Heart University, Fairfield, CT 06825, USA

**Keywords:** sweat sensor, sweat rate, dehydration, IoT, PDMS

## Abstract

There has been significant research on the physiology of sweat in the past decade, with one of the main interests being the development of a real-time hydration monitor that utilizes sweat. The contents of sweat have been known for decades; sweat provides significant information on the physiological condition of the human body. However, it is important to know the sweat rate as well, as sweat rate alters the concentration of the sweat constituents, and ultimately affects the accuracy of hydration detection. Towards this goal, a calorimetric based flow-rate detection system was built and tested to determine sweat rate in real time. The proposed sweat rate monitoring system has been validated through both controlled lab experiments (syringe pump) and human trials. An Internet of Things (IoT) platform was embedded, with the sensor using a Simblee board and Raspberry Pi. The overall prototype is capable of sending sweat rate information in real time to either a smartphone or directly to the cloud. Based on a proven theoretical concept, our overall system implementation features a pioneer device that can truly measure the rate of sweat in real time, which was tested and validated on human subjects. Our realization of the real-time sweat rate watch is capable of detecting sweat rates as low as 0.15 µL/min/cm^2^, with an average error in accuracy of 18% compared to manual sweat rate readings.

## 1. Introduction

With the popularity of smartwatches, a new lifestyle is growing, particularly in urban areas, where people are more conscious of how they treat their own bodies [1]. Numerous devices are available for professionals and exercise/technology enthusiasts that monitor body vitals such as heart rate monitors [2], temperature sensors [3], and even optical glucose measurement devices [4]. One of the growing exercise activities to stay fit and healthy is endurance sports such as marathons, biking, and triathlons [5]. Not only is there a significant increase in the number of professional athletes competing in these events, but also there has been growing interest among non-professional athletes every year in the last decade [6]. There are several smartwatches on the market that can help monitor exercise intensity and performance. Even though heart rate monitoring is considered the most effective way of monitoring the effort level, athletes are well aware that fluid intake before, during, and after a hard training is quite important [7,8]. Fluid intake of less than what is required by the body would lead to dehydration (or hypohydration), and cause both physical and mental performance degradation [8,9,10]. On the other hand, too much fluid intake, more than required (hyperhydration), can be fatal [7].

It is imperative that most of the fluid losses during a race or training occur through sweating in order to regulate the core body temperature [11]. Physical exercise leads to an increase in core body temperature, and sweating through skin cools down the body via thermoregulation. Sweat glands that are located under the skin are responsible for sweating action. Eccrine sweat glands are the main sweat glands located in human skin, and are responsible for regulating core body temperature [12]. On the other hand, apocrine sweat glands are located at armpits and groin areas that are activated during emotional and sexual arousement [13]. Although the density of eccrine sweat glands varies significantly throughout the body, some averages can be given, such as 104 glands/cm^2^ for forearms, 155 glands/cm^2^ for the forehead, etc. [14].

Research on the physiology of sweat is more than a century old, when contents of sweat were first analyzed [13]. What is in sweat is well understood; it is mostly water, with electrolytes such as sodium, potassium, urea, lactate, and other trace minerals [12,14]. Several sweat-based hydration detection monitoring systems were proposed that included optical [15,16], electrochemical [17,18], or amperometric [17,19] techniques. It is known that the amounts of individual constituents of sweat not only vary from person to person, but also show significant variations in different regions of the same individual [14,20,21]. Without establishing a base level of sweat on a particular individual’s particular body region, comparisons cannot be made. Furthermore, the concentration of sweat also changes with the rate of sweat, which is directly related to the intensity of the exercise and environmental conditions [11,22,23]. Therefore, sweat rate plays a significant role in physical conditioning, and is a useful variable for determining the performance of an athlete, particularly in endurance sports. A real-time measurement of sweat rate can ultimately provide more deterministic results on how to interpret sweat concentration data that could be used to gather physiological information about an athlete.

Sweat-rate sensing was first performed by utilizing traditional sweat collection methods such as whole-body wash [24,25] or absorbent pads [26]. Although accurate, these approaches could only provide a limited number of data points, and cannot be considered in real time. Toward sweat rate sensing, flow rate sensors were considered as an alternative. It was widely accepted that microflow sensing (flow rates around µL/min) is implemented with thermal flow rate sensors, particularly calorimetric or thermal pulse flow sensors [27,28]. Matzeu et al. demonstrated the detection of sweat rate using image analysis where they took images of the sweat filling a commercial sweat collector (Megaduct, a larger volume than a Macroduct) [29]. This approach was arguably the first real-time sweat rate detection system. However, it requires cameras and post-processing, altering the use of the system as a wearable device. Coyle et al. also proposed a preliminary sweat rate sensing textile patch employed by discrete humidity sensors [30]. Although promising, this device was only capable of measuring the evaporated sweat rather than the total secreted sweat from a region, which is called transepidermal water loss, and is not the same as the sweat rate. Wei et al. used a cellulose-based sweat absorber to measure sweat levels [31]. Real-time sweat rates were obtained, but these devices were not validated through manual measurements. Furthermore, their system relied on the weight of the absorbed sweat, which would cause hidromeiosis, i.e., the local dysfunction of sweat glands due to the blockage of sweat ducts.

Building on our years-long sweat concentration research [16,32,33,34], we have recently proposed a concept that could be used for sweat rate sensing applications, and stated that it could potentially be utilized for sweat-rate sensing applications [35]. Building upon our expertise on the Internet of Things (IoT) [36,37,38,39], we are bringing our concept into life by proposing a wearable, real-time sweat rate device. Although we have shown the feasibility of the concept in our previous work [30], this paper provides an innovative implementation of the concept, and expands the scope with human subject validation (compared with manual data collection). A unique modification in building a microfluidic calorimetric sweat rate sensor allowed us to feed the sensor with sweat via a commercial sweat collector (Macroduct). Sweat rate data was obtained, processed, analyzed, and sent to the cloud for instantaneous and longitudinal monitoring and analysis. A smartphone app was developed that displayed the sweat rate information obtained wirelessly from a LilyPad Simblee board [40]. A custom printed circuit board (PCB) was designed with surface mount components. Human trials were also conducted to validate the proposed IoT-based sweat rate monitoring system.

## 2. Materials and Methods

The flow rate sensor was built using a polydimethylsiloxane (PDMS) silicone elastomer kit (Dow Corning) by using two pieces of PDMS. A silicon wafer with a long rectangle channel mold was used. PDMS was poured into the silicon mold to create the inverse channel, which was 26 mm × 3 mm × 1 mm. Inlet and outlet holes were punched through the PDMS channel. The upper PDMS block had a 10-ohm resistor embedded in it that acted as a heater, and holes were also punched through to allow two copper–constantan T-type thermocouples (Omega, with 30 gauge wiring) to insert. Upper and lower PDMS pieces were attached together by using the plasma cleaner (Harrick PDC-32G) to form the closed channel. Thermocouples were then slid into place through the already punched holes. Loctite was used to ensure a tight seal around the thermocouples and the heating resistor.

A PCB was designed and fabricated to minimize the footprint of the electronics. A power transistor (NPN MJD200T4G) was used to supply enough current to the heating resistor. An instrumentation amplifier (AD8237ARMZ) was utilized to convert the differential temperature readings into voltage. An entire PCB was powered via the Simblee BLE’s (Bluetooth Low Energy) regulated 3.3-V output. The voltage output of the amplifier was connected to the Simblee board for wireless communication.

The characterization of two copper–constantan T-type thermocouples was done by using a Revlon turbo/lightweight/1875 W hairdryer. Thermocouples were physically kept farther apart, and one of them was heated. The process was reversed for thermocouples in order to obtain a symmetric temperature difference. The temperature reading of the thermocouples was obtained from two Omega HHM9007R multimeters (OMEGA, Norwalk, CT, USA). The voltage output of the differential temperature readings of the thermocouples was obtained from the instrumentation amplifier. A non-zero reference voltage was used for the amplifier. The temperature value and corresponding output voltage were then recorded.

The characterization of the sweat rate sensor device was performed by pumping deionized (DI) water through the channel with a syringe pump (KDS Scientific 100) at flow rates from 3 µL/min to 100 µL/min, which were equally spaced logarithmically. The temperatures and the output voltage of the amplifier were recorded once the flow rate reached a steady state value (about 10 min).

The Macroduct sweat collector (ELITechGroup Biomedical Systems, South Logan, UT, USA) was used to guide the sweat to the PDMS channel. A 3.7-V 500 mAh rechargeable LiPo PKCell battery was used to power the Simblee board, which then powered the PCB. The entire device was housed in a box that was custom designed using TinkerCAD (Version 3.9, Autodesk, CA, USA), and three-dimensional (3D) printed by Makerbot Replicator + (New York City, NY, USA).

Human trials were conducted using an exercise bike. In order to record the sweat rate sensor manually, a Macroduct was placed on the left upper forearm of the sweat rate sensor prototype, and the volume of sweat in the Macroduct tubing was recorded over time. The manual rate of sweat was then calculated by taking into account the dimensions of the Macroduct tubing. The sweat rate sensing device was put on the right upper forearm. Output voltages of the device were wirelessly transmitted to a smartphone (HTC One M7 WLS and iPhone 6s) via the LilyPad Simblee BLE board, and a mobile app was utilized to read the data every 15 s or 30 s. A cloud interface was built around ThingSpeak. A Raspberry Pi 3 was used as the receiver and connection to the cloud. An online stopwatch (Free-stopwatch.com) was used to keep time accurately for the sweat rate testing. Five subjects were tested after they gave informed consent. Institutional Review Board (IRB) approval was obtained at Sacred Heart University (IRB #170922A). Subjects were asked to bike on the exercise bike at 80% workload of their maximum heart rate, which was calculated as 208 − (0.7 × age). Room temperature was 24 °C, and the relative humidity was recorded as 40%. Subjects were allowed to drink water before, during, and after ad libitum.

## 3. Device Architecture

**Figure 1:**
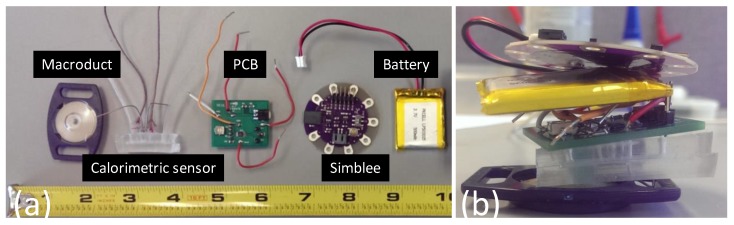
Components of the sweat rate sensing device: (**a**) Individual parts that were used to build the prototype; (**b**) overall device view after the assembly and connections were made.

The device was built on the commercial Macroduct sweat collector with some modifications. The coiled tubing of the Macroduct was shortened to 1 cm, and the tubing was connected to the inlet of the calorimetric sweat rate sensor to guide the sweat into the sweat rate sensing channel. Temperature information readings, as well as heating element control circuitry, were designed and custom built on a PCB. Information gathered from the sensor was processed at the PCB electronics, and the voltage output of the sweat rate information was sent to the Simblee board for wireless transmission. The circuitry was powered by the Simblee board, which had a LiPo battery as its own power supply. The individual components of the entire system and the assembled device photos are shown in Figure 1a,b, respectively.

**Figure 2:**
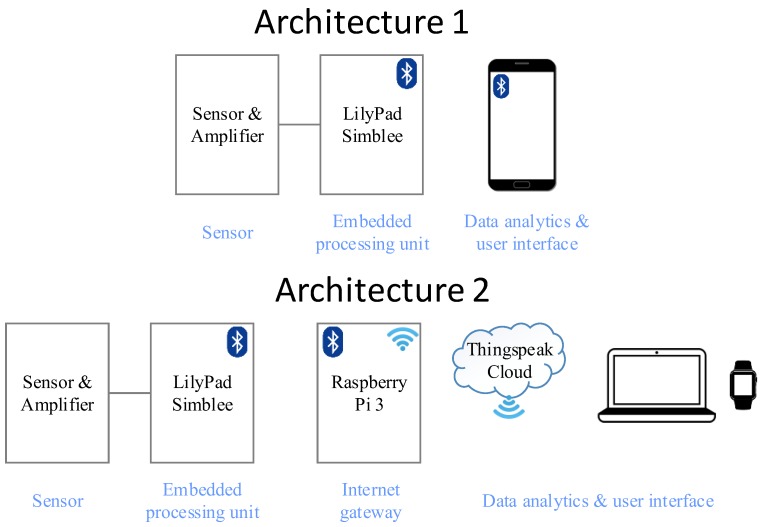
Architectural components of an Internet of Things (IoT) framework for instantaneous (architecture 1) and longitudinal (architecture 2) sweat rate analysis.

Two system architectures have been developed to test the sweat rate sensor’s IoT functionality, as illustrated in Figure 2. Both architectures are compatible with the current system, and no hardware modifications needed to be made on the device. The first architecture purely focuses on monitoring the instantaneous sweat rate of just one user, and provides a warning to the user so that appropriate actions can be taken when the level goes beyond a threshold value. This monitoring is accomplished through a cell phone application with which the LilyPad Simblee can directly communicate. The second architecture is designed such that the sweat rate from multiple users could be obtained simultaneously, longitudinal data analytics could be performed, and data could be stored for further analysis by a clinical expert. This architecture requires an internet gateway, which was implemented by a Raspberry Pi 3 on the receiver side. A ThingSpeak IoT platform was utilized for data analytics, storage, and visualization.

## 4. Results

**Figure 3:**
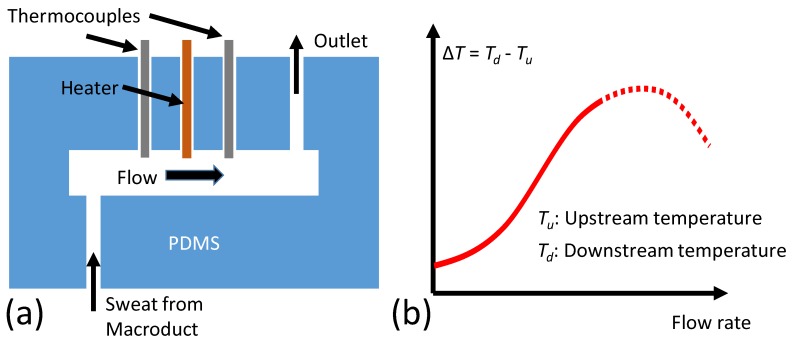
Theoretical principles of the calorimetric flow rate device. (**a**) Two thermocouples (upstream and downstream) were placed along the heating element. Sweat entered the channel from the bottom, and exited from the top as an outlet; (**b**) The temperature difference between thermocouple readings increased with the flow and reached a maximum. Realistic flow rates were well below this maximum. The temperature difference is a monotonously increasing function of the flow rate.

Figure 3a shows the initial sweat rate sensing concept where the initial theoretical operation was introduced by the authors [30]. A heating element is placed in a flow channel. Two thermocouples (upstream and downstream) are placed on each side of the heater (2-mm spacing) to sense the temperature. Once there is a flow, temperature around the upstream sensor (*T_u_*) cools off because of the fluid flow. Temperature around the downstream sensor (*T_d_*), on the other hand, increases with the flow as the temperature profile is pushed further with the flow. Once a critical flow rate value is reached (detailed theoretical analysis can be found in [31]), *T_d_* starts to cool off as well (due to high flow rates). The temperature difference, Δ*T* = *T_d_* − *T_u_*, reaches a maximum at this critical flow rate, as illustrated in Figure 1b. In practice, this threshold occurs at high flow rates that are well beyond the human sweat rate. Therefore, a more realistic region of Δ*T* was represented with a solid line, and the theoretical (but not practical) extrapolation of Δ*T* is shown as the dashed line in Figure 2b.

**Figure 4:**
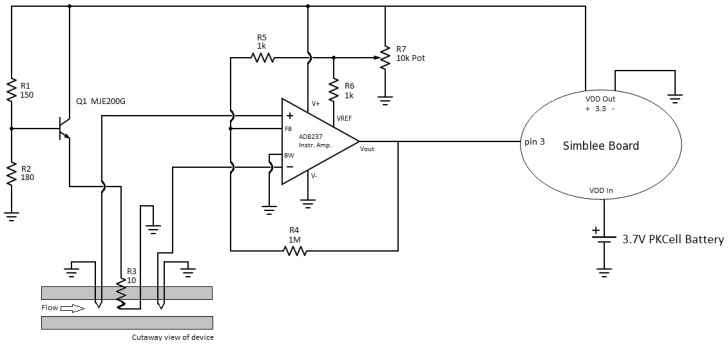
Schematic diagram of the IoT platform. A power transistor was used to set the current to the heating resistor. The temperature difference was converted into voltage via the instrumentation amplifier, and fed to the Simblee board for wireless communication.

The overall circuit diagram of the sensor and its electronics was provided in Figure 4. The base voltage of a transistor (NPN MJD200T4G) is biased using a simple voltage divider. The current that the transistor draws flows through a 10-ohm resistor connected between the emitter and the ground. This resistor acts as a heater, and is embedded in the PDMS device. The temperature upstream and downstream of the heater are both sensed by copper–constantan T-type thermocouples (Omega). The thermocouples produce a voltage depending on the temperature with a slope of 40 µV/°C. Both of the thermocouples are inputs to a linear instrumentation amplifier (AD8237ARMZ), which takes the difference between the two and amplifies the result to obtain voltage values that can be detected by the microcontroller. External resistors set the gain of the amplifier, and the reference voltage is set by a 10-kΩ potentiometer. The output voltage of the amplifier is then sent to the Simblee board for data conversion and wireless transfer. The Simblee board has a regulated 3.3-V power supply that powers the transistor network, as well as the amplifier. An external LiPo 3.7 V 500 mAh PKCell battery powers the Simblee board.

**Figure 5:**
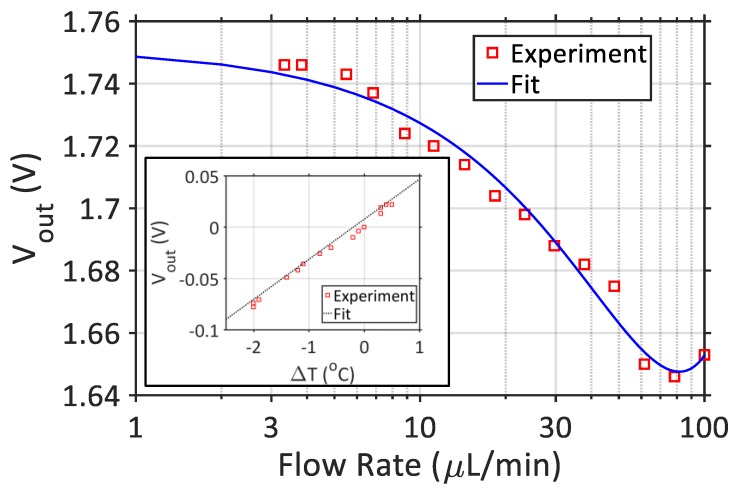
Characterization of the IoT device. The inset shows the linear relationship between the output voltage and the temperature difference. The main figure shows the output voltages that were obtained from different flow rates (generated with a syringe pump). A quadratic polynomial was fit to the data so that the output voltages can be converted into flow rate in real human trials.

The circuitry was first tested by changing the temperature around the thermocouples using a hairdryer to characterize the temperature to voltage conversion. It can be seen from the Figure 5 inset that a linear voltage increase occurs with temperature changes. In this experiment, ∆*T* was changed on a range of approximately 4 °C to show the trend. By setting the instrumentation amplifier gain to 1000, and knowing that the thermocouple voltage conversion is 40 µV/°C, an ideal function can be derived as *V_out_* = 40 µV/°C × 1000 × ∆*T* + *V_ref_*, where *V_ref_* represents the reference voltage for the amplifier. The reference voltage in Figure 5 inset was subtracted for visualization purposes.

Once the circuitry operation has been validated, the sweat rate device was characterized by using a syringe pump for various volumetric flow rates (in µL/min). Figure 5 shows the output voltage values with respect to varying flow rates, which were aligned with the theoretical illustration in Figure 2b. It can be seen that *V_out_* starts to increase back from the minimum point at around 80 µL/min, which would make detection difficult (two flow rate data points would correspond to the same voltage value). On the other hand, flow rates below 1 µL/min do not result in a significant change in output voltage, reducing the resolution of the measurement. Therefore, the volumetric flow rate detection limit is determined to be between 1–80 µL/min. It is important here to note that the sweat rate is given in flow rates per unit area (cm^2^). Although these experiments were conducted by pushing the liquid to the PDMS channel directly from a syringe pump, corresponding sweat rate values could be calculated by using the area of the Macroduct, which would feed the sweat to the sensing area. As the diameter of the Macroduct is 2.7 cm, the minimum sweat rate that can be detected with the current system would be 0.15 µL/min/cm^2^. Although the upper limit of detection for the device is calculated to be around 13 µL/min/cm^2^, this limit would never be reached, because it is known that the sweat rate for humans does not exceed 5 µL/min/cm^2^. Hence, the range of the device fits well to the application. A second order polynomial was fit to the experimental data, which can be used to obtain the flow rate (FR) from the output voltage (*V_out_*).

**Figure 6:**
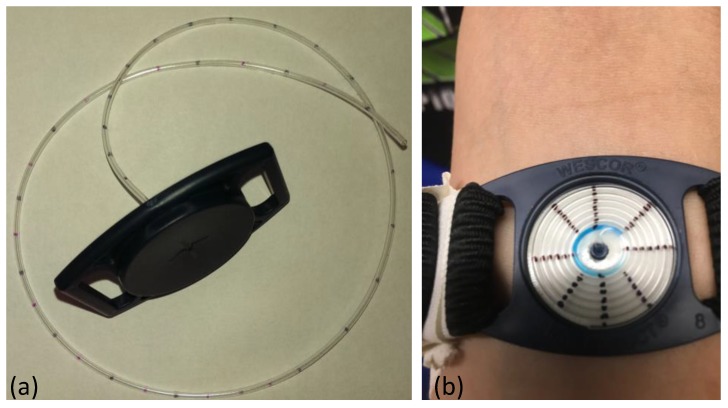
Manual determination of the sweat rate was achieved with a Macroduct sweat collector. (**a**) Uncoiled Macroduct tube to measure the distance between each mark; (**b**) Photo of the Macroduct being filled with the sweat during an actual subject trial.

In order to validate the sweat rate device, human subject trials were conducted. A commercial Macroduct was used as the control. The Macroduct collects the sweat in the spiral tubing as the collector makes contact with the skin, and the small pinhole at the bottom allows the sweat to go through the tube. Spiral Macroduct tubing was marked every 45° turn, which corresponds to a 5-mm distance between each mark. The exact locations of the marks were determined by uncoiling the spiral tube, as shown in Figure 6a. The volume of the sweat between each mark was calculated using the inner diameter of the tubing (0.64 mm). The time it took the sweat to go from one mark to another was noted, and the volumetric flow rate was calculated accordingly. This method allowed us to record sweat rate values approximately every 2 µL in the tube. The Macroduct tube has a dye in it that made it easier to visualize the location of the sweat. An actual Macroduct usage on a subject is shown in Figure 6b.

Two male and three female participants completed the exercise protocol (Table 1). Exercise duration ranged from 18 min to 30 min, while the average sweat rate was 0.76 ± 0.41 µL/min/cm^2^. Pre and post-weights were measured with clothes. Therefore, weight loss during the exercise only provides a qualitative measure, and can be assumed that the overall weight loss was greater. No fluids were ingested during the trial. In order to provide more quality information regarding the validation of the device, error values in accuracy were devised from the data by calculating the percent deviation of the sweat rate (*SR*) from the device data (*SR*_device_) to the manual Macroduct collection (*SR*_control_), and are presented in the Table 1 as the percentage error (calculated as $100x\left(SR_{device}-SR_{control}\right)/SR_{control}$). The percent error in accuracy for all of the subjects ranged from 4–30% (Table 1).

**Figure 7:**
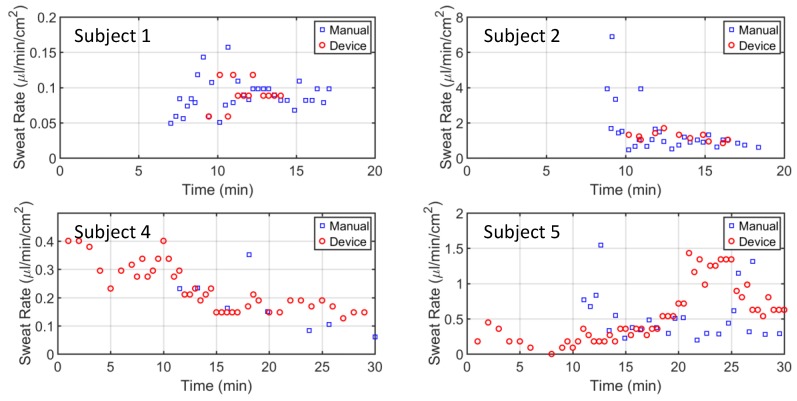
Human trials were conducted to validate the sweat rate sensing device. Manual data collection from the Macroduct was recorded by visually observing the sweat going through the spiral tube. Sweat rate from the device was recorded from the Smartphone.

The sweat rate device was worn on the opposite arm to the manual data collection, and data was collected simultaneously. Sweat rate readings were gathered every 30 s from the phone app. Figure 7 shows four of the five subject tests. It is apparent that the manual readings from the Macroduct collection started 7–10 min after the exercise started, as it takes time for the Macroduct reservoir to begin filling. Similarly, device data for subjects 1 and 2 were also delayed. Subject 4 and 5 data from the device started from the very beginning, due to the residual DI water left from rinsing the device. This residual water was left in the tube intentionally to lower the latency of the device. As expected, subjects’ sweat rate patterns varied during the exercise protocol, with most of the patterns being relatively constant after 12 min of exercise.

**Figure 8:**
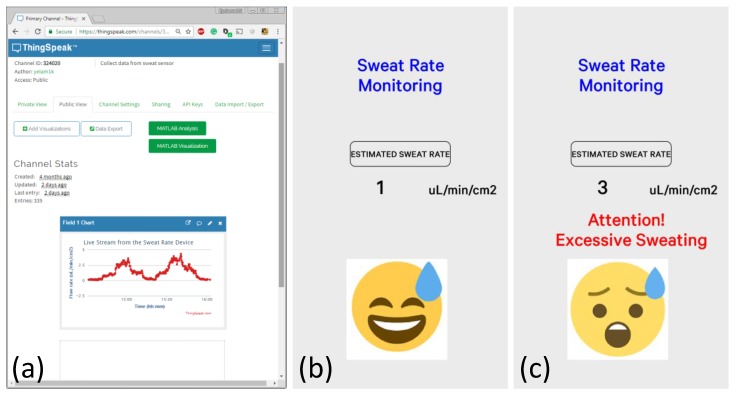
The IoT framework with a sweat rate sensor prototype. (**a**) The ThingSpeak web interface was used for real-time monitoring of the data; (**b**,**c**) The smartphone app was developed based on the Simblee’s interface to give instantaneous feedback to the user.

The IoT framework was tested with the actual sweat rate sensor platform. Figure 8a shows the real-time data of the sweat rate sensor, where different flow rates were introduced to the device. The flow rate was calculated from the obtained voltage values using the second-degree polynomial obtained earlier. ThingSpeak offers a great way of post-processing the data using embedded MATLAB functions (as seen in green buttons). The mobile app, as shown in Figure 8b,c, was designed to give instantaneous feedback to the user if the sweat rate values reached excessively high levels. Currently, this threshold was set to 1.5 µL/min/cm^2^, which can easily be adjusted depending on the situation. If the threshold is exceeded, the app gives a warning (shown in Figure 8c) on the screen.

## 5. Discussion

The current device has a size of 6 cm × 6 cm × 5 cm, which equates to a form factor of 180 cm^3^. Considering that the form factor of a sports watch is around 25 cm^3^, the size of the current sweat rate sensing device is big. From the end product point of view, the PDMS device can be made much thinner, and a smaller battery could be used. Lastly, a PCB can be embedded into the PDMS. These efforts could potentially reduce the form factor to below 100 cm^3^, which would be acceptable for commercialization.

The overall current consumption of the device is around 80 mA. Most of the current is consumed at the heating element. The Simblee board is capable of providing enough current to the device. The current consumption of the instrumentation amplifier is 0.1 mA, which can be considered negligible. During the data transmission, the Simblee board can consume as high as 10 mA. The relatively large current consumption of the entire device results in the need for a high-capacity battery. If the current is lowered on the heating element, the temperature differences, and hence the output voltage swings, get significantly smaller, limiting the sensitivity of the conversion. One way to reduce the current consumption is to implement the heater using thin films, i.e., microfabrication techniques. This approach would significantly lower the form factor and allow the device to become more sensitive at below 0.15 µL/min/cm^2^. The authors had theoretically proved that the effective range of the device can be shifted to lower flow rates by downscaling the device dimensions, particularly the distance between the heater and the respective thermocouples [35].

In order for the Macroduct sweat collector to start filling up the coiled tube, the interface between the skin and the Macroduct needs to fill first. However, because the Macroduct conforms to the skin, we assumed this volume is less than 1 µL. There is about 1 cm of coiled tube before the sweat enters the sweat-sensing channel, resulting about 4 µL of sweat that is not analyzed. Furthermore, both of the thermocouples that are in the sweat channel need to be immersed in sweat. Considering this distance is around 1 cm (from the location where the sweat enters the channel to the downstream thermocouple), and the cross-section of the channel is 3 mm × 1 mm, potentially 30 µL of sweat would be required. Overall, almost 35 µL of sweat would be needed to start receiving sweat rate data. An average sweat volume rate of 3 µL/min (approximately 0.5 µL/min/cm^2^) would result in a 10-min latency, which is consistent with our observations for subjects 1 and 2 (Figure 7). However, the sweat channel and Macroduct tubing can be filled with DI water from PDMS outlet in order to reduce the latency. It can be seen from subjects 4 and 5 (Figure 7) that the latency was completely removed.

It is apparent that the local sweat rate cannot be a good representative of the whole body sweat rate. However, a rough estimate can be performed. Taylor et al. provided a detailed study on regional sweat rate variations [14]. In their seminal work, morphologically normalized and referenced adult values (weight: 70 kg, height, 1.7 m, body surface area, BSA: 1.8 m^2^) were defined, and the active sweat gland density for the forearm was given as 104 glands/cm^2^ [14]. The total number of active eccrine sweat glands on the entire body was estimated to be around two million [14]. An average of 1 µL/min/cm^2^ of local sweat on the forearm then would result in a 0.4 L (~0.4 kg) of sweat loss in 20 min. Our preliminary weight loss measurements revealed that subjects lost at least 0.2–0.3 kg during the exercise. Although these numbers are approximations, it is evident that the values are within the same scale.

The average error in the accuracy of the sweat rate device was found to be 18%. However, it must be noted here that manual data collection with the Macroduct also presents inherent challenges in terms of its own accuracy. The time it takes the sweat to travel from one marked location on the tube to the next was visually observed, which could easily yield data-recording errors. Also, each Macroduct was attached to forearms using stretchable bands. The amount of pressure that is applied to the skin can also affect the functionality of both data collection methods.

All of the subsystems that were used in the IoT framework were off-the-shelf components with open-source software libraries available, and minimal effort would be required if the system were to be modified for a different analysis.

## 6. Conclusions

We have presented a wireless real-time sweat rate sensing platform for athlete hydration detection applications. A calorimetric flow rate sensor was utilized in combination with a Macroduct sweat collector to detect the rate of sweat. Data collection was carried out by establishing an IoT framework through either: (1) displaying the sweat rate information on a smartphone app, or (2) displaying on the cloud via ThingSpeak. Controlled flow rate characterization was performed using a syringe pump to calibrate the device. Human subject trials were conducted to validate the device.

The lower limit of sweat rate detection for the current device is around 0.15 µL/min/cm^2^. Considering that the device was developed for athletes who would sweat more due to heavy exercise, this limit could potentially not be a practical limit in field tests. For example, one of our subjects (subject 4) was a very light sweater, but we were still able to gather her sweat rate information via our device.

The theoretical concept of the calorimetric flow rate system was investigated by our group [36]. Our current work significantly expanded on this knowledge, and we built a standalone real-time sweat rate device. Our implementation of combining the Macroduct sweat collector with the calorimetric sweat rate sensor (inlet from the bottom, and outlet from the top by using a double PDMS mold) is novel. Furthermore, the device was validated with five subjects, resulting in an average error in accuracy of 18%, which is considered to be very promising. Our device is unique in approach and validation, which separates us from camera-recorded sweat rate measurements [29] or the absorbent patch approach [30,31] that was discussed earlier.

We believe the current developments on IoT interfaces and physiological sensor devices will lead to cloud-based health awareness and tracking tools for unique exercise routines. Our current approach will help pave the way for wireless, real-time sweat-based hydration systems.

## Figures and Tables

**Table 1:** Results from the validation of the sweat rate sensor device.

Subject #	Gender	Pre Test Weight (kg)	Weight Loss (kg)	Duration (min)	Sweat Rate (µL/min/cm^2^)	Error (%)
1	Female	62.4	>0.2	18	0.91	30
2	Male	93.9	>0.3	20	1.21	16
3	Male	75.6	>0.3	20	0.98	15
4	Female	52.7	>0.2	30	0.17	4
5	Female	76.0	>0.2	30	0.54	27
